# Defining the influence of size‐exclusion chromatography fraction window and ultrafiltration column choice on extracellular vesicle recovery in a skeletal muscle model

**DOI:** 10.1002/jex2.85

**Published:** 2023-04-21

**Authors:** María Fernández‐Rhodes, Bahman Adlou, Soraya Williams, Rebecca Lees, Ben Peacock, Dimitri Aubert, Aveen R. Jalal, Mark P. Lewis, Owen G. Davies

**Affiliations:** ^1^ School of Sport, Exercise and Health Sciences Loughborough University Loughborough Leicestershire UK; ^2^ NanoFCM Co., LTD Nottingham Nottinghamshire UK

**Keywords:** extracellular vesicles, isolation, size‐exclusion chromatography, skeletal muscle, ultrafiltration

## Abstract

Extracellular vesicles (EVs) have the potential to provide new insights into skeletal muscle (SM) physiology and pathophysiology. However, current isolation protocols often do not eliminate co‐isolated components such as lipoproteins and RNA binding proteins that could confound outcomes and hinder downstream clinical translation. In this study, we validated an EV isolation protocol that combined size‐exclusion chromatography (SEC) with ultrafiltration (UF) to increase sample throughput, scalability and purity, while providing the very first analysis of the effects of UF column choice and fraction window on EV recovery. C2C12 myotube conditioned medium was pre‐concentrated using either Amicon^®^ Ultra 15 or Vivaspin^®^20 100 KDa UF columns and processed by SEC (IZON, qEV 70 nm). The resulting thirty fractions obtained were individually analysed to identify an optimal fraction window for EV recovery. The EV marker TSG101 could be detected from fractions 5 to 14, while CD9 and Annexin A2 only up to fraction 6. ApoA1^+^ lipoprotein co‐isolates were detected from fraction 6 onwards for both protocols. Strikingly, Amicon and Vivaspin UF concentration protocols led to qualitative and quantitative variations in EV marker profiles and purity. Eliminating lipoprotein co‐isolation by reducing the SEC fraction window resulted in a net loss of particles, but increased measures of sample purity and had only a negligible impact on the presence of EV marker proteins. In conclusion, our study developed an effective UF+SEC protocol for the isolation of EVs based on sample purity (fractions 1–5) and total EV abundance (fractions 2–10). We provide evidence to demonstrate that the choice of UF column can affect the composition of the resulting EV preparation and needs to be considered when being applied in EV isolation studies in SM. The resulting protocols will be valuable in isolating highly pure EV preparations for applications in a range of therapeutic and diagnostic studies.

## 1 INTRODUCTION

Extracellular vesicles (EVs) are a diverse family of nanosized particles delimited by a lipid bilayer. Their cargo includes proteins (e.g., oncogenic regulators and transcription factors), nucleic acids (e.g., mRNA, microRNA, Y RNAs), lipids and metabolites that are delivered to recipient cells after activation of host cells surface‐receptors or by fusion with the recipient plasma membrane (Mulcahy et al., 2014), leading to phenotypic changes (Heath et al., 2018; Popovic, 2019). EVs can be broadly separated into three subpopulations based on their cellular origin and biogenesis. These include exosomes (size range ∼30‐150 nm), microvesicles (MVs) (size range ∼100‐1000 nm) and apoptotic bodies (size range ∼0.5‐2 µm). Due to their overlapping sizes and bio‐compositions, effectively differentiating between the three groups is challenging and it is recommended that they are collectively termed EVs (Théry et al., 2018). These particles are produced by practically every cell type in the human body and have diverse and often complex functions in a range of physiological and pathophysiological processes such as tissue homeostasis, regeneration and inflammation (El Andaloussi et al., 2013; Harrell et al., 2019; Lawson et al., 2016; Takov et al., 2019; Yáñez‐Mó et al., 2015). This has led to growing interest in their application in healthcare as diagnostic biomarkers of pathologies including cancers, bone disease or autoimmune diseases (Dai et al., 2021; Ruan et al., 2021; Zhang et al., 2021). However, the roles of EVs in skeletal muscle (SM) development, regeneration and ageing are less well defined due to a lack of an optimised isolation method offering a highly pure and high throughput means of isolation.

SM is primarily characterised by its mechanical functions. It is formed through the activation and differentiation of resident satellite cells to myoblasts, their subsequent fusion to form myotubes and their aggregation into myofibers. Over the past two decades, the secretory role of this tissue and the contribution of myokines in autocrine, paracrine and endocrine processes has been well described (Severinsen & Pedersen, 2020). However, the contribution of EVs in these processes remains much less well defined. Historically, EVs were first isolated in SM studies from undifferentiated C2C12 cells by Guescini et al., who predicted their role in the modulation of IGF‐1 signalling and intra‐ and inter‐organ communication through proteomic analysis (Guescini et al., 2010). Since this initial study, EV‐enriched fractions have been isolated from immortalised C2C12 cell lines at different stages of the myogenic differentiation process, as well as from primary myoblasts, myotubes and murine SM fibres (Forterre et al., 2014; Romancino et al., 2013). However, due to the lack of availability of bespoke protocols for the isolation of EVs from SM, the majority of publications to date have studied EV‐enriched fractions that also contain additional co‐isolated particles such as lipoproteins and protein aggregates (Obi et al., 2022; Rome, 2022; Rome et al., 2019). Consequently, while outcomes from previous studies have provided highly interesting observations concerning the potential functions of EVs in process such as myogenic regulation and energy metabolism (Kim et al., 2018; Shuler et al., 2020; Sork et al., 2018; Takafuji et al., 2020), they have frequently not accounted for the presence and contribution of lipoproteins and RNA binding proteins (e.g., AGO2) commonly recovered in EV preparations, which due to their overlapping diameters (low density lipoproteins: 20–200 nm) and densities (high density lipoproteins: 1.06–1.21 g/mL), can lead to inaccuracies if not depleted in the isolation protocol (Yuana et al., 2014). Additionally, HDLs are known carriers of RNAs, which could lead to potentially false conclusions that miRNA biomarkers are associated with EVs (Tabet et al., 2014). This not only poses considerable issues in the accurate identification of EVs but also risks the potential mislabelling or overrepresentation of EVs as delivery vehicles for established and emerging myokines and exercise (Safdar et al., 2016; Trovato et al., 2019).

Within the SM field, the majority of in vitro studies have applied differential centrifugation (dUC) and density gradient centrifugation (Baci et al., 2020; Davies et al., 2021; Forterre et al., 2014; Guescini et al., 2015, 2010; Kim et al., 2018; Le Bihan et al., 2012; Romancino et al., 2013; Sork et al., 2018; Takafuji et al., 2020; Vumbaca et al., 2021; Q. Xu et al., 2018) or commercial isolation kits (Hettinger et al., 2021; Le Gall et al., 2020; Shuler et al., 2020; Q. Xu et al., 2018) for the isolation of EVs. In vivo, studies have applied dUC (Annibalini et al., 2019; Guescini et al., 2015; Kawao et al., 2018; Mitchell et al., 2019; Picca et al., 2020), polyethylene glycol (PEG) (Fulzele et al., 2019) or commercial isolation kits (Fry et al., 2017; Mytidou et al., 2021; Vechetti et al., 2021). These techniques are becoming increasingly challenged by emergence of physically milder isolation methods including size‐exclusion chromatography (SEC), which can enhance sample purity that is frequently defined by particle‐to‐protein (PTP) ratio (Busatto et al., 2018; Böing et al., 2014; Heath et al., 2018; Lobb et al., 2015; Welton et al., 2015, Webber & Clayton, 2013). SEC represents an increasingly utilised method for EV isolation—with a 30% marked increase in the number of publications since 2016 (Gardiner et al., 2016; Royo et al., 2020; Sidhom et al., 2020). This method allows for the recovery of multiple fractions that can independently be tested for the presence of EVs and lipoproteins to optimise the purity of the final recovery window or the possibility of automatization for increasing sample uniformity. However, SEC may display some limitations, such as the recovery of dilute low volume EV preparations. To date, SEC has been only minimally applied for the isolation of EVs from SM, with only one example of this method being applied in cell culture to monitor miRNA stability in C2C12 cells (Coenen‐Stass et al., 2019). While two studies have applied SEC for the isolation of EVs from blood samples to monitor changes in miRNAs associated with exercise (Kobayashi et al., 2021; Lovett et al., 2018). However, in these examples, the authors provided no conclusive validation of EV containing fractions in accordance with published guidelines (Lötvall et al., 2014; Théry et al., 2018). Furthermore, in order to increase throughput and sample purity (PTP and reduced lipoprotein content), SEC is often combined with an ultrafiltration (UF) step (Kornilov et al., 2018; Nordin et al., 2015; Parimon et al., 2018; R. Xu et al., 2015). Different UF systems are available that differ in membrane composition and molecular weight cut‐off (MWCO), with columns being manufactured from materials including regenerated cellulose (Amicon) and polyethersulfone (PES, Vivaspin). Membrane composition and MWCO are likely to have a significant effect on EV yield but have been only minimally examined in the scientific literature and, to the best of our knowledge, not for EVs isolated from SM (Nordin et al., 2015; Vergauwen et al., 2017). For non‐SM sources, the choice UF column and SEC fraction windows varies throughout the literature (Benedikter et al., 2017; Bertoldi et al., 2017; Karimi et al., 2018; Kratzer et al., 2016; Takov et al., 2019; Vergauwen et al., 2017) and it is becoming increasingly clear that these parameters need to be optimised for each cell type and starting fluid in order to maximise EV recovery and sample purity. However, no study has yet sought to optimise a combined UF+SEC protocol for the high‐throughput isolation of comparatively pure EV from SM preparations. The optimisation of such a system will be critical if we are to accurately define SM‐EV biomarkers that can provide new insights into SM physiology and pathophysiology.

This study sought to critically define the effect of UF column and SEC fraction for the isolation of highly pure EVs from C2C12 SM cells. To achieve this, we compared EV recovery from two widely utilised UF columns (Amicon® Ultra 15 (100 kDa) and Vivaspin®20 (100 kDa)), identifying an optimal SEC fraction window to enhance sample purity for prospective biomarker studies.

## 2 MATERIALS AND METHODS

### 2.1 Cell culture

**FIGURE 1:**
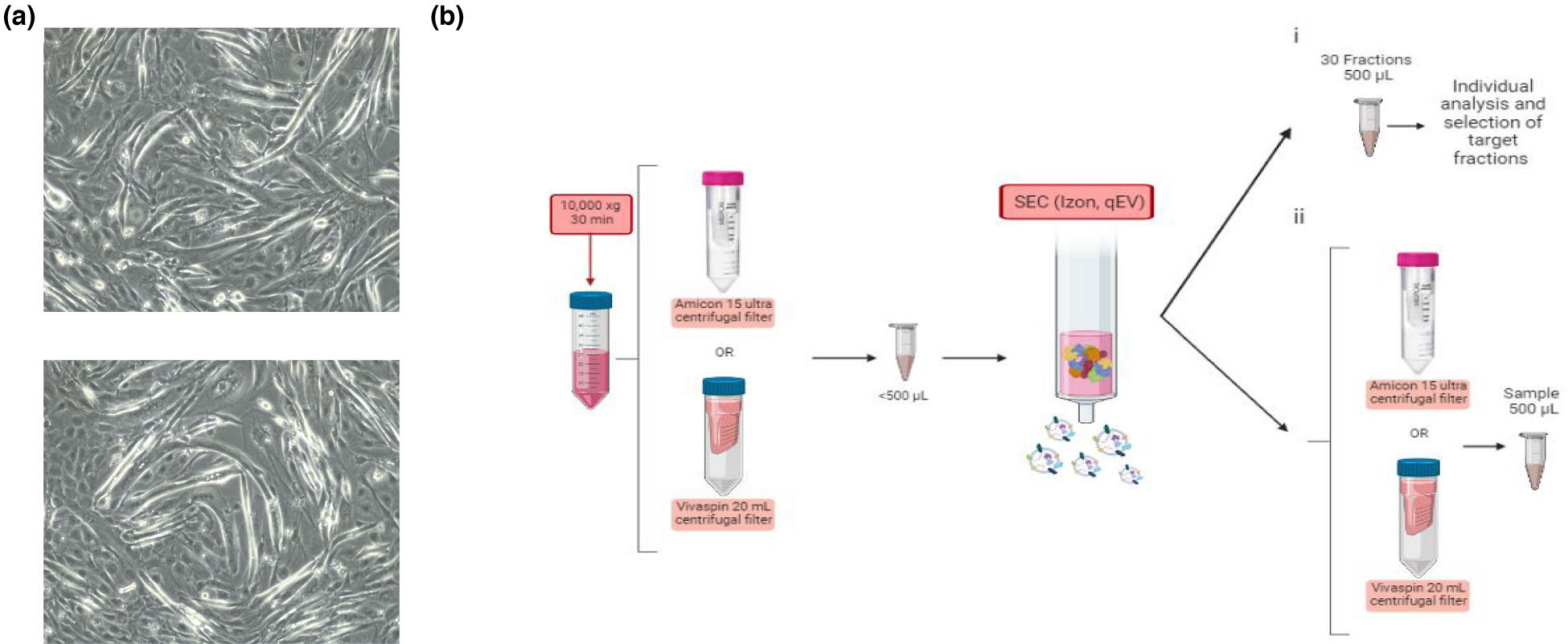
Workflow for EVs isolation by size‐exclusion chromatography. (a) Light microscopy images of C2C12 cells at 48 h of differentiation (b) conditioned medium from C2C12 skeletal muscle cells was concentrated using Amicon or Vivaspin UF columns following the manufacturer's instructions and processed using SEC (i) where 30 individual fractions were collected for independent analysis or (ii) further UF concentrated and combined to generate fraction windows for downstream comparison. Created using Biorender (https://biorender.com/).

C2C12 murine SM myoblast (*ATCC® CRL‐1772™*) cells were grown using standard growth medium (GM) composed of high glucose Dulbecco's Modified Eagle's Medium (DMEM) (*D6429, Merck KgaA, Darmstadt, Germany*), 20% fetal bovine serum (FBS) (*P40‐37500, Pan Biotech, UK*), and 1% Penicillin/Steptomyocin (P/S) (*11548876, FisherScientific, UK*). 1 × 10^6^ cells were cultured in T175 flasks (*12034917, FisherScientific, UK*) and incubated in a 5% CO_2_ humidified atmosphere at 37°C until 80% confluence was attained. After reaching 80% confluence, the growth media was replaced with differentiation media composed of DMEM High Glucose, 2% horse serum (HS) largely depleted in EVs (*30021200, Pan Biotech, UK*) and 1% P/S. HS was depleted of EVs by spinning at 120,000 × *g* for 16 h. After 48 h differentiation (Figure 1a) conditioned media (CM) was collected and centrifuged at 2000 × *g* for 20 min to remove cells and debris (*Heraeus Megafuge 11, rotor TX‐1000, ThermoFisher, UK*). 20 mL of CM was collected per repeat and stored at −80°C up to 3 months for EV isolation.

### 2.2 EV isolation

CM (*N* = 3) was first spun at 10,000 × *g* for 30 min (*himac CS‐150NX, rotor S50A, Hitachi, Japan*) and then concentrated using Amicon^®^ Ultra 15 (100 kDa) *(UFC910024*, *Merck KgaA, Darmstadt, Germany*) or Vivaspin®20 (100 kDa) (*GE28‐9323‐63, Merck KgaA, Darmstadt, Germany*) UF columns according to the manufacturer's recommendations (*Heraeus Megafuge 11, rotor TX‐1000, ThermoFisher, UK*). Concentrated sample was submitted to SEC columns (qEVoriginal/70 nm, IZON SCIENCE LTD, New Zealand), collecting 30 independent fractions of 500 µL using an Automatic Fraction Collector (AFC) (IZON SCIENCE LTD, New Zealand) (Figure 1b(i)). In instances where 500 µL fractions were combined to maximise EV recovery, where both UF devices were reapplied to reduce total sample to equal volumes of 500 µL (Figure 1b(ii)).

### 2.3 Nanoparticle tracking analysis (NTA)

Size distribution and particle concentration for each sample were measured using the NanoSight LM10 *(Malvern Panalytical, UK)*. EV fractions were diluted using DPBS *(15326239, FisherScientific, UK)*, capturing measurements of 20–100 particles per frame, using three different batches. Six videos with a duration of 30 s each were captured of each sample. Settings for videos and data acquisition were constant with camera level set between 13 and 15 and screen gain between 1 and 2 and using a laser with 405 nm wavelength. All runs were analysed with the same threshold. Data was analysed using Nanosight NTA 3.2 software (*Malvern Panalytical, UK*).

### 2.4 Bicinchoninic acid (BCA) protein assay

BCA assay was applied to determine protein concentration for each fraction used. Pierce™ BCA Protein Assay Kit (*23227, ThermoFisher Scientifics, UK*) was used according to the supplier's instructions. 25 µL of each sample, neat or diluted (1:2) was loaded in a 96‐well plate, followed by 200 µL of the BCA/copper complex solution from the kit. The absorbance was measured at 562 nm in the microplate reader Thermo Scientific Varioskan Flash *(ThermoFisher Scientifics, UK)* using Skanlt software (*SkanIt Software 2.4.5 RE*).

### 2.5 Western blot (WB)

Samples at 1 µg/mL were prepared adding 25% of Sample buffer (SB4X) *(1610747, BioRad, UK)* and lysis buffer (LB) [0.5% Triton X‐100, EDTA 1X and protease inhibitors *(10085973, FisherScientific, UK)] to complete volumes*. Samples were boiled for 5 min at 98°C. Proteins present in the samples were separated in precast polyacrylamide *gels (4561083, BioRad, UK)*, loading 5 µg protein and using three replicates per sample in all cases. Precision Plus Protein™ Dual Color Standards were applied for estimation of molecular weight *(1610374, BioRad, UK)*. Protein bands were transferred to Polyvinylidene fluoride (PVDF) membranes *(11544996, FisherScientific, UK)* that were blocked in EveryBlot blocking buffer *(12010020, BioRad, UK)* and washed in Tris buffer solution with 0.1% Tween20 *(Merck™ 655204‐100ML, FisherScientifics, UK)* (TBST). Membranes were incubated with primary antibodies (Supplementary Table 1) overnight at 4°C. The following day, membranes were washed three times and incubated by lightly shaking with the appropriate secondary antibody for 1 h at room temperature (Supplementary Table 1). Protein bands were detected using the ChemiDoc XRS+ system *(1708265, BioRad, UK)* and Image Lab 3.0.1 software *(Life Science Research, BioRad, UK)*. Image J 1.46 (*National Institutes of Health, USA*) was applied for WB band quantification.

### 2.6 Nano flow cytometry (nFCM)

nFCM was performed using a NanoAnalyzer U30 instrument (*NanoFCM Inc., Nottingham, UK*). The nFCM instrument was equipped with photon‐counting avalanche photodiodes (APDs) and two dual 488/640 nm lasers that were applied for simultaneous detection of side scatter (SSC) and fluorescence‐based detection of individual particles. All samples were diluted to 1.87 × 10^10^ particles/mL and stained with CD9‐FITC (1:200) (*124809, BioLegend, UK*), CD63‐APC (1:100) *(ab233056, abcam, UK*) or CD81‐APC (1:100) (*104909, BioLegend, UK*) for 30 min at room temperature. Optical alignment was tested and calibrated using fluorescent 250 nm silica nanoparticles. Further calibration measurements were taken prior to analysis using 250 nm silica nanoparticles of known concentration (for EV concentration calculation) and the proprietary four‐modal silica nanosphere cocktail generated by NanoFCM containing nanosphere populations of 68, 91, 113, and 155 nm diameter (for EVs size calculation). nFCM analysed samples by gating particles ranging from 40 to 200 nm. Samples were measured at least three times and data was processed by nFCM Professional Suite v1.8 software, using a gating for subpopulations separated by fluorescent intensities with size distribution and concentration available for each sub‐population.

### 2.7 Zeta potential measurements

Zetasizer Nano ZS (*Malvern Panalytical, UK*) was used to perform Z‐potential measurements. DTS1070 folded capillary cells were washed with isopropanol and ionized water and then dried before applying the sample. Once cleaned, samples were submitted to the capillary cell and device (*Malvern Panalytical, UK*). Measurement time was 60 s at room temperature in Monomodal mode at 50 mV.

### 2.8 Transmission electron microscopy (TEM)

EVs were prepared on continuous carbon (Cu 300 mesh) TEM grids. Samples were fixed in glutaraldehyde 3% and, once dry, washed twice with milliQ water. To visualise EVs, slides were negatively stained with 1% Uranyl Acetate and air dried. Images were recorded on an FEI Tecnai G2 12 Biotwin TEM (*ThermoFisher, USA*) operating at 100 kV, using a Gatan SIS Megaview iV camera (*EMSIS GmbH, Germany*).

### 2.9 Statistical analysis

Graphs were prepared using Origin Lab (*OriginLab Corporation, USA*), with values presented as mean ± standard deviations (SDs) with 95% confidence intervals. Pearson's correlation‐r was used for correlations within samples. Student's t‐tests (two tailed tests) and analysis of variance (ANOVA) with Bonferroni post‐hoc were performed using GraphPad Prism 6 (*GraphPad Software, San Diego, USA*). Differences were considered statistically significant at *p < 0.05, **p < 0.01, or ***p < 0.001

## 3 RESULTS

### 3.1 EV recovery

**FIGURE 2:**
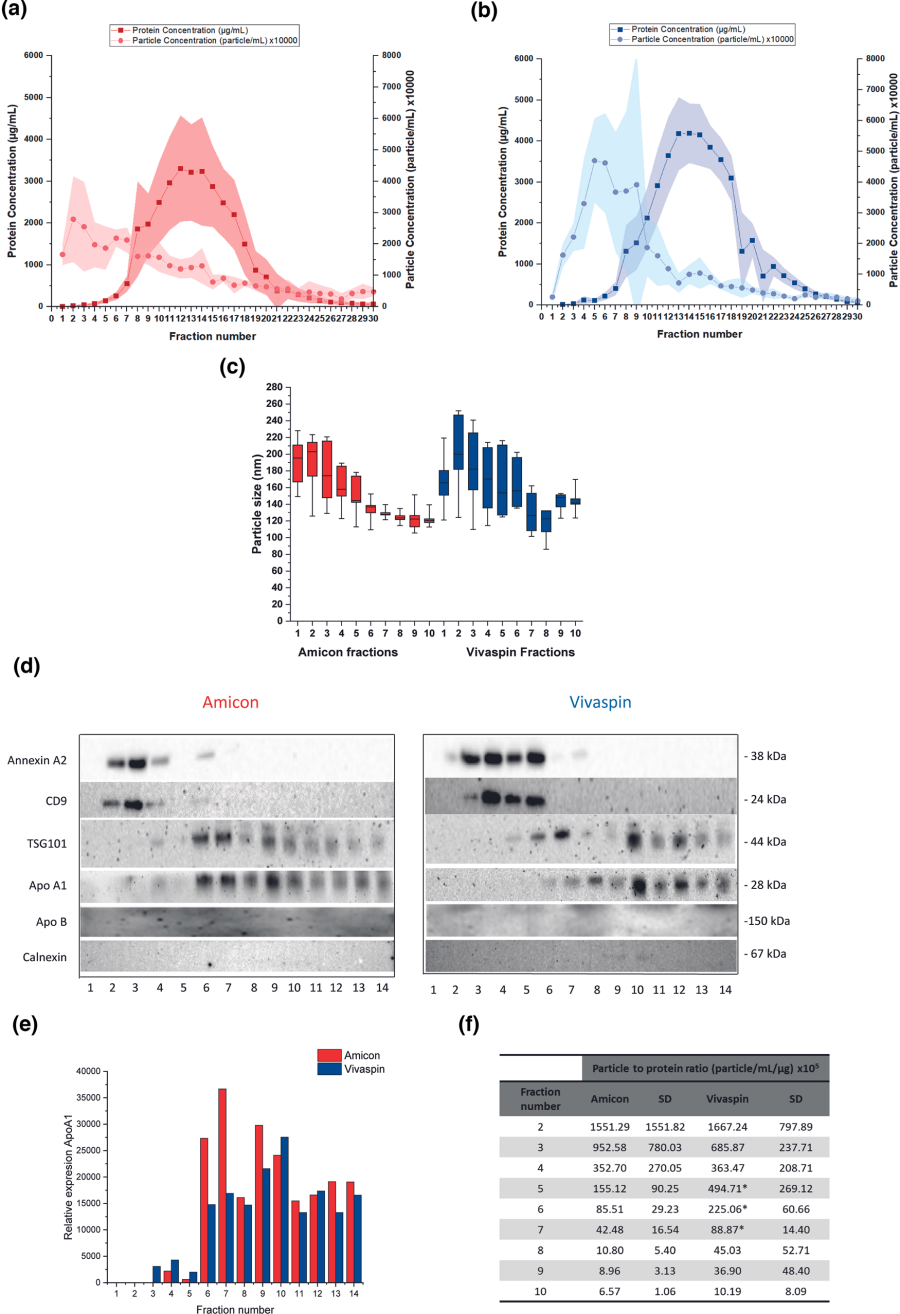
Analysis of 30 individual SEC fractions following Amicon and Vivaspin UF pre‐concentration (*N* = 3). (a) Representation of particle and protein concentration per fraction when using Amicon filters; light red, particle component; dark red, protein component. (b) Representation of particle and protein concentration per fraction when using Vivaspin filters; light blue, particle component; dark blue, protein component. (c) Fraction size distributions for Amicon and Vivaspin. We highlighted just the measurements within the first 10 fractions. (d) Analysis by WB of the presence of EVs and non‐EVs markers across the first 14 fractions when pre‐concentrating with Vivaspin or Amicon filter devices. The presence of EVs is confirmed by CD9, Annexin A2 and TSG101. (e) Relative presence of ApoA1 across fractions 1–14 in Amicon (red) and Vivaspin (blue) pre‐concentrated samples. Graphs were obtained after the quantification of the protein detected on each band using ImageJ software and eliminating minimum background and expression in the extracted image. (f) PTP ratios (particle/mL/protein concentration in mg/mL) related to the first 10 fractions for both isolation methods.

In order to understand which fractions would be included in the combined EV preparation, 30 independent fractions were collected from differentiated C2C12 cultures following CM pre‐concentration (Figure 1b(i)). Total protein concentrations began to elevate considerably between fraction 7 and 8 for both UF protocols (three‐fold changes for both methods respectively, *p* < 0.05). A decline in particle concentration intersected with an increased total protein concentration between fractions 8 and 10 when using Amicon and Vivaspin filters, respectively (Figures 2a,b and Supplementary Figure S1). Peak of particle concentration was found within the first 14 fractions. Fraction 5 seemed to be the peak for Amicon + SEC isolation protocol, although when using Vivapsin filters, the highest particle concentration was detected between fractions 5 and 6. Particle size distributions obtained by NTA measurements, reflecting the mode size ± SD, were 76.30 ± 3.00 versus 93.43 ± 3.4 nm and mean size ± SD: 158.15 ± 19.33 versus 163.41 ± 30.12 nm, for Amicon and Vivaspin, respectively (Figures 2c and Supplementary Figure S1). Broader size distribution of particles was encountered in Vivaspin fractions, especially between fractions 2 and 7 (Figures 2b,c and Supplementary Figure S1). 72% and 85% of particles were measured in fractions 1–10 for Amicon and Vivaspin columns, respectively (Figures 2a,b and Supplementary Figure S1). Qualitative detection of positive (Annexin A2, TSG101 and CD9) and negative EVs markers (Calnexin, ApoA1 and ApoB) by WB showed marker profile differences within all fraction's windows analysed. CD9 and Annexin A2 were detected in fractions 2 to 4, but with some presence in fraction 6, following Amicon pre‐concentration, although, in fractions 3 to 6 and 2 to 6 for Vivaspin pre‐concentrated samples. TSG101 showed positive signal between fractions 6 to 14 when using Amicon but in fractions 5 to 14 after Vivaspin filtration (Figure 2d). Presence of EV tetraspanins CD63 and CD81 were analysed by ExoELISA assay, showing an increased signal from fraction 4 (Supplementary Figure S2a,b) confirming the enrichment of EVs within that range of fractions for both isolation protocols. High‐ (ApoA1) and low‐density (ApoB) lipoprotein markers were used to provide an indication of the relative purity of each fraction. Of the negative markers analysed, Calnexin and ApoB could not be detected in any samples analysed. ApoA1 was visibly increased from fraction 6 after Vivaspin and Amicon pre‐concentration (Figure 2d,e). When evaluating PTP ratio, as an indirect measure of sample purity, fractions 5, 6, and 7 in Vivaspin pre‐concentrated samples fractions had a significantly higher value compared to the samples obtained following Amicon pre‐concentration (Figure 2f).

### 3.2 Maximising EV recovery: Analysis of EV‐enriched fractions (2–10) following UF pre‐concentration

**FIGURE 3:**
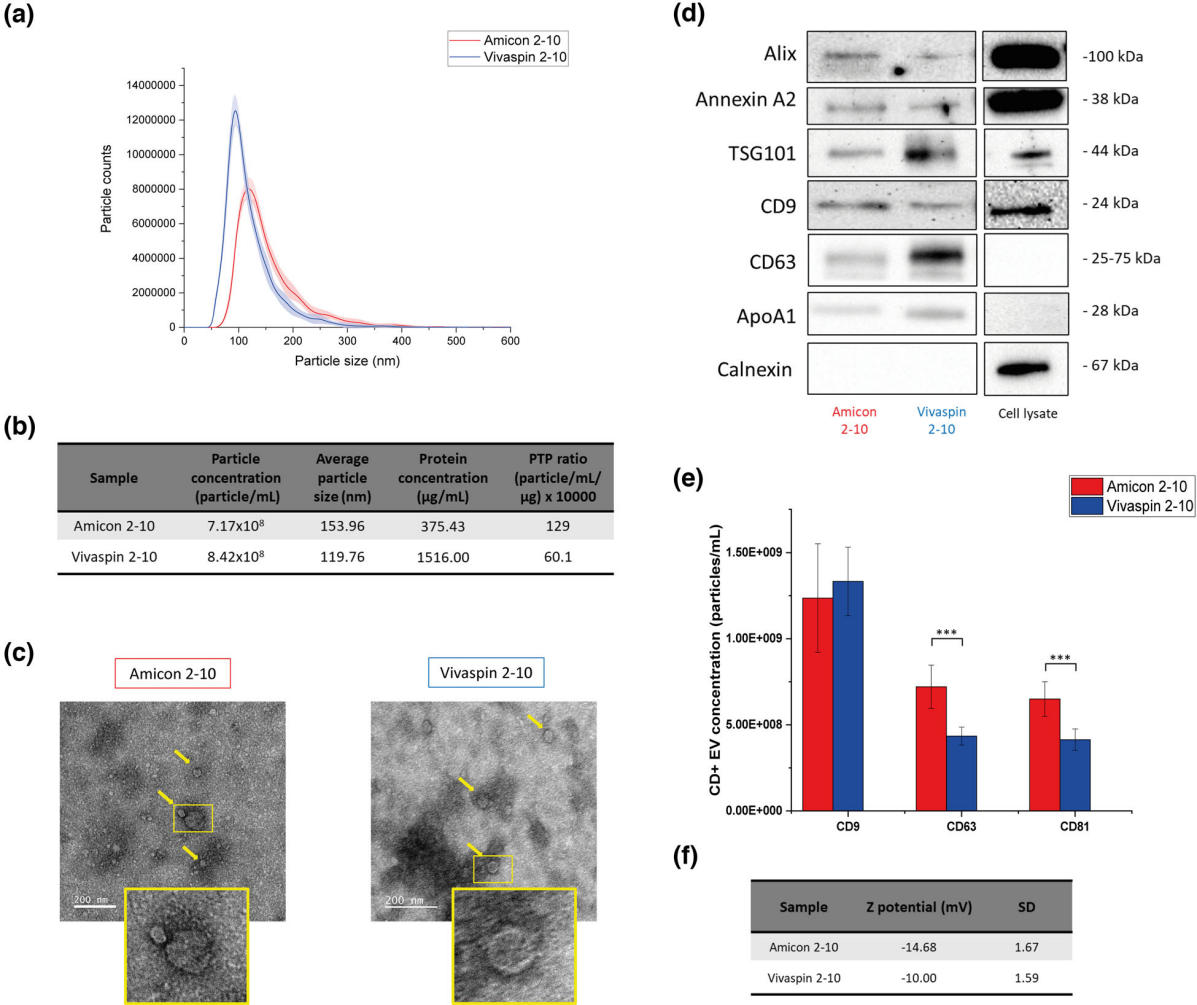
Analysis of combined and re‐concentrated samples from fractions 2–10 by Amicon or Vivaspin UF devices (*N* = 3). (a) Particle size distributions of Amicon (red) and Vivaspin (blue) pre‐concentrated samples. SD are indicated in the figure as a shadow depending on the colour of each representing sample. (b) Purity characterisation. Average particle and protein concentrations, size and PTP of the three samples. (c) TEM images. Obtained from final concentrated samples. Yellow arrows point to stained EVs. (d) Evaluation of EVs markers by Western blot. Results comparison between Amicon and Vivaspin 2–10 pre‐concentrated samples. Cell lysate was included in the analysis to test the presence of ER related content. (e) Specific detection of EV‐like particles. nFCM measurements of the presence of CD9, CD63, and CD81 for both isolation protocols. (f) Z potential (mV) measurements. Samples were measured using DPBS as buffer.

Based on the outcomes of Figure 2, an initial fraction window of 2–10 was selected for both UF columns to provide comparable yield of EV enriched material while minimising—but not fully eliminating—lipoprotein contamination at this stage (Figure 1b(ii)). Within this combined fraction window, Vivaspin columns recovered 15% more particles than Amicon columns, with particle concentrations of 8.42 × 10^8^ and 7.17 × 10^8^ particles/mL, respectively (Figure 3a,c). Particles recovered after Amicon pre‐concentration had a wider size distribution compared to Vivaspin UF + SEC protocol (Figure 3a), and larger particles were isolated using Amicon columns (mean size ± SD: 153.96 ± 12.31 nm; mode size ± SD: 99.83 ± 11.85 nm; *p* > 0.05) when compared to Vivaspin (mean size ± SD: 119.76 ± 15.30 nm; mode size ± SD: 90.00 ± 11.27 nm; *p* > 0.05) (Figure 3a,b). Total protein concentration was significantly (*p* < 0.05) reduced for Amicon preparations (Figure 3b). This trend was reflected in the PTP ratios, where higher purity was recorded following Amicon pre‐concentration (129.0 vs. 60.1). TEM analysis of samples provided visual evidence of individual SM‐EVs for both isolation protocols, which could be visually identified by the presence of a cup shaped morphology (Figures 3c and Supplementary Figure S3). WB analysis revealed qualitative differences in marker presence between the two UF + SEC protocols, with TSG101 and CD63 most abundant in Vivaspin fractions and CD9 and Alix most abundant in the Amicon fractions. Calnexin was absent from all UF + SEC preparations and only visible in the cell lysate control. ApoA1 was detected in both UF + SEC preparations, but more predominantly in Vivaspin preparations (Figure 3d). Data provided by nFCM measurements showed that in particles sized 40–200 nm, CD9 was the most abundant tetraspanin in both isolations (Figure 3e), but the qualitative differences showed for this marker by WB (Figure 3d) were not supported. nFCM measurements also revealed significantly (*p* < 0.001) higher concentration of CD63^+^ and CD81^+^ particles were detected in the samples obtained after Amicon pre‐concentration when compared to Vivaspin (Figure 3e). Finally, zeta potential measurements identified values of −14.68 mV and −10 mV following Amicon and Vivaspin UF respectively, with no significant differences between them, but more negative values were detected in Amicon preparations (*p* > 0.05) (Figure 3f).

### 3.3 Refining fraction window to exclude lipoproteins

**FIGURE 4:**
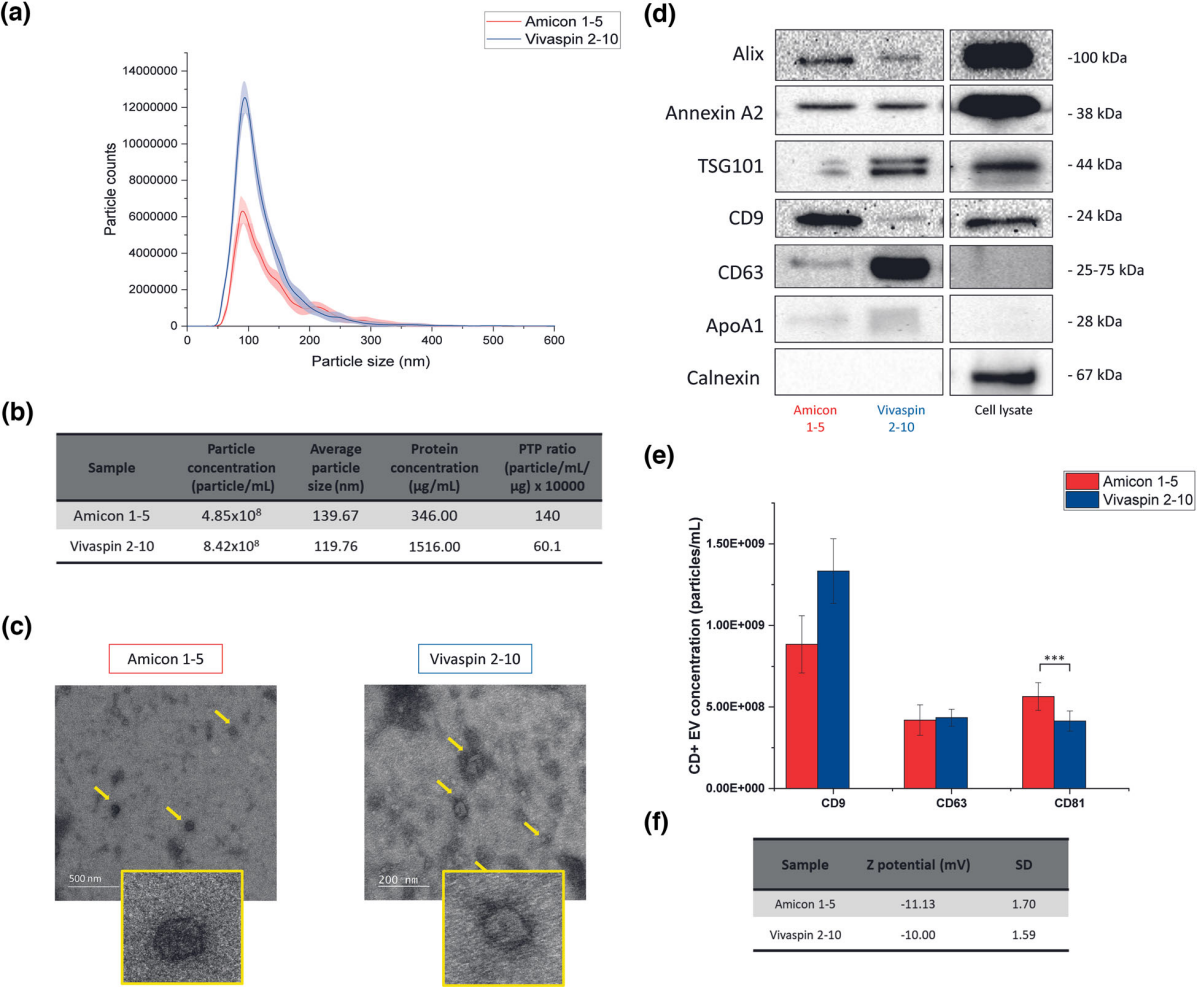
Analysis of combined and re‐concentrated samples from fractions 1–5 Amicon or 2–10 Vivaspin UF devices (*N* = 3). (a) Particle size distributions of Amicon (red) and Vivaspin (blue) pre‐concentrated samples. SD are indicated in the figure as a shadow depending on the colour of each representing sample. (b) Purity characterisation. Average particle and protein concentrations, size and PTP of the three samples. (c) TEM images. Obtained from final concentrated samples. Yellow arrows point to stained EVs. (d) Evaluation of EVs markers by Western blot. Results comparison between Amicon 1–5 and Vivaspin 2–10 pre‐concentrated samples. Cell lysate was included in the analysis to test the presence of ER related content. (e) Specific detection of EV‐like particles. nFCM measurements of the presence of CD9, CD63, and CD81 for the different isolation protocols. (f) Z potential (mV) measurements. Samples were measured using DPBS as buffer.

In Figure 2, we highlighted an increased presence of lipoproteins beyond fraction 6 after pre‐concentrating with both UF protocols. For this part of this research, fractions 1–5 obtained after Amicon pre‐ and post‐concentration were compared to Vivaspin pooled fractions 2–10 previously analysed (Figure 1b(ii) and 3) to observe the effects of eliminating fractions enriched in lipoproteins on EV recovery. Total particle concentration was reduced to 4.85 × 10^8^ particles/mL for samples pre‐concentrated using Amicon filters (Figure 4a,b). This finding appeared to be visually supported by the TEM analysis in which fewer particles could be observed per frame (Figure 4c and Supplementary Figure S3). Average particle sizes of 139.67 ± 12.31 nm were recorded for pooled Amicon fractions 1–5, while mean sizes ± SD of 153.96 ± 12.31 nm (mode size ± SD: 92.48 ± 0.97 nm) were recorded for Vivaspin preparations, however without any significant differences (*p* > 0.05) (Figure 4a,b). PTP ratio increased from 129 (Figure 4b) to 140 (*p* > 0.05) when combining fractions 1 to 5 compared to fractions 2 to 10 following Amicon UF and Vivaspin preparation PTP ratio was again lower (Figure 4b). The presence of EV specific markers was qualitatively examined by WB (Figure 4d), revealing that the overall trend in marker expression was not dissimilar to data presented in previous results (Figure 3d). The WB data highlighted an increased presence in CD9 and Alix for Amicon pooled fractions 1–5, but not TSG101 and CD63 (Figure 4d). Quantitative comparison of EV markers revealed significant increases in CD81^+^ particles were maintained when reducing the fraction window following Amicon pre‐concentration relative to Vivaspin fractions 2–10 (*p* < 0.001) (Figure 4e). However, significant differences in CD63 could no longer be observed when compared with Vivaspin fractions 2–10 (Figure 4d,e). Zeta potential measurements were −11.13 mV and −10.00 in Amicon and Vivaspin preparations respectively, observing a decrease in this value for Amicon preparations containing fractions 1–5 compared to the Amicon 2–10 preparation (*p* > 0.05) (Figure 4f).

### 3.4 Selection of protocol depending on downstream applications

**FIGURE 5:**
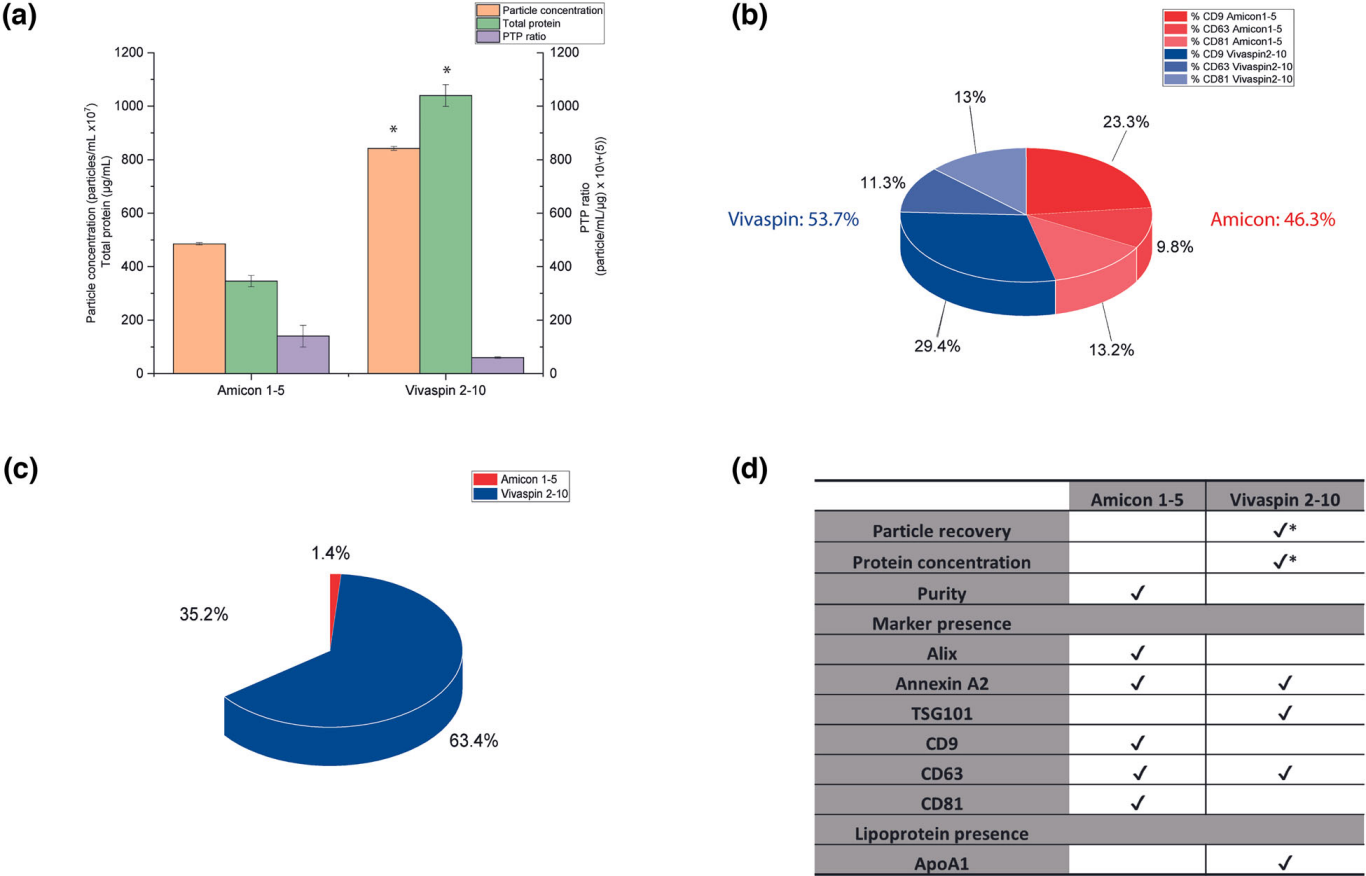
Comparison of EV preparations recovered using Amicon (fractions 1–5) and Vivaspin (fractions 2–10) UF + SEC. (a) Representation of sample purity. Particle (left Y axis) and protein concentrations and PTP ratio (right Y axis), representing purity for both Amicon 1–5 and Vivaspin 2–10 preparations. (b) Main tetraspanins presence (%). CD9, CD63, and CD81 nFCM measurements showed in and *4*. (c) Lipoprotein band quantification. Representation of WB bands quantifications by ImageJ. (d) Summary table indicating the main outcomes to select the isolation protocol of choice. Method criteria for marker presence was based on WB detection differences supported by nFCM data where appropriate. Different outcomes and characteristics of the isolation protocols described earlier might influence the application of each technique.

We will now summarise the results to demonstrate how UF  +  SEC can be applied to enhance EV purity (fractions 1–5) or total particle recovery (fractions 2–10). Protein and particle concentrations were significantly higher (*p* < 0.05) in Vivaspin 2–10 preparations. However, PTP ratios were 57% higher for Amicon fractions 1–5 final products (*p* > 0.05) (Figure 5a). When comparing the outcomes of reducing the Amicon fraction window (fractions 1–5), a reduction of the 32% was observed in particle content. While only a reduction of 8% in total protein concentration and PTP ratio was observed in this case (*p* > 0.05) (Figure 5a). No significant differences were recorded for the combined presence of tetraspanin proteins CD9, CD63, and CD81 as recorded using nFCM for total (46.3% total recovery for Amicon 1–5 and 53.7% total recovery for Vivaspin 2–10) or individual (23.3% for CD9, 9.8% for CD63 and 13.2% for CD81 in Amicon 1–5 preparations versus 29.4% for CD9, 11.3% for CD63 and 13% for CD81 in Vivaspin preparations) EV tetraspanins recovery, as determined using nFCM (*p* < 0.001) (Figure 5b). We demonstrated almost complete elimination of lipoprotein co‐isolates (1.4%) in the Amicon 1–5 preparations, in contrast to Vivaspin 2–10 preparations (63.4%) (Figure 5c). Lastly, the table provided (Figure 5d) gives a summary of outcomes of interest, which is designed to enable other researchers to select the SEC + UF protocol most suitable for their own applications. Results suggested that in order to maximise purity, combination fractions 1–5 after Amicon pre‐concentration would be the most suitable approach, but that Vivaspin fractions 2–10 could be utilised to maximise EV recovery at the expense of sample purity. From here, differences in EV marker presence have been highlighted. TSG101 and CD63 were consistently increased for Vivaspin preparations, while CD9 was the predominant marker for Amicon 1–5 preparations. Finally, greater presence of CD81^+^ particles was encountered in Amicon 1–5 preparations but no differences were observed regarding the CD63^+^ population (Figure 5d and Supplementary Figure S3).

## 4 DISCUSSION

No optimal method has been developed for the isolation of EVs from SM. To date, studies have applied multiple isolation methods that often lack specificity, such as dUC (Baci et al., 2020; Davies et al., 2021; Forterre et al., 2014; Guescini et al., 2010; Kim et al., 2018; Le Bihan et al., 2012; Maeshige et al., 2021; Romancino et al., 2013; Sork et al., 2018; Takafuji et al., 2020; Vumbaca et al., 2021; Q. Xu et al., 2018), PEG or commercial isolation kits (Hettinger et al., 2021; Le Gall et al., 2020; Shuler et al., 2020; Q. Xu et al., 2018). While neutral on a functional level, any EV isolation protocol can affect sample quality, introducing impurities or a variety of co‐isolated particles (lipoproteins and protein/RNA complexes) (Baranyai et al., 2015; Taylor & Shah, 2015; Zhao et al., 2022). Not only does this have a confounding effect on therapeutic studies, it can also risk the elimination or aggregation of EVs, thereby masking potentially valuable biomarkers (J. Lee et al., 2021; Ludwig et al., 2018; Seo et al., 2020). These factors make distinguishing the precise origin of EVs in highly complex biofluids and cell cultures media containing platelet lysates or residual serum components extremely challenging (Estrada et al., 2020). Furthermore, HDLs found in biofluids and cell cultures are also carriers of RNAs, which unless depleted in the EV preparation can lead to potentially false impressions that miRNA biomarkers are associated with EVs (Tabet et al., 2014). SM in particular is a highly communicative tissue, with secreted proteins, such as myokines, being released in addition to EVs. Secreted EVs from SM in vitro models have been associated with prominent muscle markers and myokines, such as, myosine heavy chain (MyHC) and desmin (Forterre et al., 2014; Le Bihan et al., 2012). Furthermore, 50%–80% of the reported myokines were listed as proteins or peptides in plasma secreted EVs (Safdar & Tarnopolsky, 2018). However, these reports relied principally on mass spectrometry observations of peptide fragments recovered from EV preparations using isolation methods such as UC or commercial isolation kits. To the best of our knowledge, further evidence of a direct association between these myokines and EVs is required to substantiate reports of their localisation. These considerations not only pose considerable issues in the accurate identification and separation of EVs in SM samples, but also risks the potential mislabelling or overrepresentation of EVs as delivery vehicles for established and emerging myokines and exercines (Safdar et al., 2016; Trovato et al., 2019). As such, there exists a requirement to define optimal isolation protocols for the purification and profiling of EVs in defined in vitro SM systems before biomarker studies can be effectively progressed in complex biofluids. It is imperative that isolation protocols are developed to reduce lipoprotein contaminants to a minimum to enable the comprehensive and accurate characterisation of SM‐EVs. In the present study we tested an isolation methodology combining SEC + UF for the recovery of EV fractions from a C2C12 cell line. Using a pure SM cell system, we were able to validate a specific fraction window for the collection or EVs and elimination of lipoprotein contaminants. Lastly, we were able to identify the impact of often indiscriminately applied UF pre‐concentration on EV recovery.

SEC has been shown to be a promising high‐throughput and adaptable method for the isolation of EVs from a range of biofluids such as plasma and urine, as well as cell culture medium (Lobb et al., 2015; Lozano‐Ramos et al., 2015; Stranska et al., 2018). However, since SEC recovers multiple fractions, it is important that each is independently analysed to monitor the presence of EVs and co‐isolated lipoproteins to optimise a final recovery window for a given application. Additional contamination factors, such as other lipids or metabolites, were not accounted for in our research. Currently, soluble proteins or miRNAs are the most studied contaminants alongside lipoproteins in in vitro (Böing et al., 2014; Coenen‐Stass et al., 2019; Pavani et al., 2020) and in vivo research (Karvinen et al., 2022; Kobayashi et al., 2021; Maggio et al., 2023). Future studies will be needed to better identify the extent of non‐lipoprotein contaminants in EV preparations, particularly for SM applications. In the outcomes of the present study, we have identified distinct fraction windows able to enhance EV purity (fractions 1–5 after Amicon UF) and recovery (fractions 2–10 for both isolation protocols). Identifying an appropriate fraction window for EV collection is a major consideration when applying SEC, as this can differ depending on the source material (e.g., cell culture medium, plasma, urine, etc.) and cell/tissue type (Karimi et al., 2018). Large variations (SDs) were found between repeats for each fraction (Figure 2). This could result from the fact that the NTA method applied for the analysis of independent fractions cannot accurately distinguish authentic EVs from co‐isolated particles of similar sizes (e.g., lipoproteins). This was observation is perhaps best reflected for Vivaspin isolations, where fractions 5–10 contained notable lipoprotein contamination, as distinguished by the presence of ApoA1 (Figure 2b). Following fraction pooling and additional UF concentration, these variations in particle concentration were reduced (Figures 3 and 4). Additionally, the inclusion of NanoFCM and ExoELISA was able to provide a comparatively robust assessment of EV content based on defined surface markers. This allowed us to accurately distinguish variability in EV enriched fractions isolated from our SM model. To date, relatively few studies have applied SEC to obtain EVs from SM myoblasts. In a study by Coenen‐Stass et al. 2019, the selection of EV enriched fractions was based on absorbance at 280 nm, representing total protein content of the samples, where they observed a peak from fractions 4 to 9. Grouping these fractions they encountered particles with a modal size of 125 nm and the presence of EV markers ALIX, TSG101, and CD81 (Coenen‐Stass et al., 2019). Soluble proteins, such as enzymes, were separated, however, no mention of other common co‐isolates, such as lipoproteins, was reported. Our study reflected similar outcomes in separation, particle size and marker positivity, although we revealed the presence of ApoA1^+^ lipoproteins within fractions 6–10 in the final preparations. ApoA1 is a marker of HDL and, as previously indicated, are relevant in miRNAs circulation and signalling (Tabet et al., 2014). As such, it remains to be determined whether miRNAs identified in the previous studies are truly associated with EVs or the result of co‐isolated particulates. PEG or commercial isolation kits based on precipitation (ExoQuick and Total Exosome Isolation Kit) have also been applied in studies seeking to isolate and characterise EVs from SM. While interesting observations have been recorded for EVs derived from human primary skeletal cells cultured ex vivo, such as induction of myogenic differentiation and regeneration activities (Choi et al., 2016; Hettinger et al., 2021; Shuler et al., 2020), these precipitation‐based isolation methods introduce multiple external contamination from the source. For this reason, the application of one step precipitation methodologies is broadly discouraged within the literature (Lobb et al., 2015; Stam et al., 2021; Taylor & Shah, 2015; Théry et al., 2018).

Lipoprotein contamination in EV preparations represents a considerable limitation if we wish to accurately determine the physiological functions of SM‐EVs and develop EV diagnostics for the predication of pathological outcomes related to SM ageing and degeneration. The metabolism of lipoproteins has long been shown to be influenced by endurance training and training‐induced adaptations in SM (Kiens & Lithell, 1989) and it has long been appreciated that exercise training has a notable effect on the relative numbers of lipoproteins within the circulation (Wharton & Nustad, 2006). Nonetheless, the majority of exercise studies often do not seek to differentiate variation in EVs from those of lipoproteins. As such, it is possible that variations in circulating nanoparticles reported in exercise studies could partly be explained basic variations in non‐EV components and not specifically EVs (Sódar et al., 2016). Indeed, while multiple studies have demonstrated interesting variations in the presence of circulating nanoparticles in human and animal acute exercise models, they have often utilised non‐specific isolation methods such as dUC without testing for the presence of lipoproteins (Frühbeis et al., 2015; Whitham et al., 2018). dUC was the first method applied for the isolation of EVs from SM and remains the most broadly used (Forterre et al., 2014; Guescini et al., 2010; Le Bihan et al., 2012; Maeshige et al., 2021; Romancino et al., 2013; Rome et al., 2019; Sork et al., 2018; Takafuji et al., 2020; Q. Xu et al., 2018). dUC has been applied to isolate EVs from SM for downstream proteomic analysis, in which its comparison against a commercial isolation kit showed improvements in EV yield and purity (Le Gall et al., 2020). In this instance, polymer‐based isolation, combined with clean‐up steps using 100 kDa Amicon filters improved the isolation of functional EVs. However, the influence of lipoproteins was once again not reported, likely underestimating their role and influence in EV functionality. This is also true for an ex vivo study where dUC was applied to isolate EVs from murine SM explants. In this study EVs from obese mice presented altered lipid profiles and miRNAs targeting fatty acid metabolism pathways (Jalabert et al., 2021). However, as with the previous study, lipoprotein recovery was not assessed even though lipoproteins have been implicated in the transport of miRNAs and this could lead to erroneous reporting of EVs in RNA dynamics (Li et al., 2018; Vickers et al., 2011). Similar limitations can also be observed in EVs obtained from in vivo SM studies. In such studies, plasma has been the predominant source used (Conkright et al., 2022; Estrada et al., 2022; Lovett et al., 2018; Warnier et al., 2022). Results have reflected an increase in EV production after activity, increase of some exercise related miRNAs (miR‐1, 133a, 133b, 206, and 486) (Lovett et al., 2018) and changes in the SM‐EV tetraspanin profile (Conkright et al., 2022; Estrada et al., 2022; Warnier et al., 2022). However, just one of the studies (Warnier et al., 2022) accounted for the presence of lipoproteins in their EV samples. In contrast to our results, Warnier et al. claimed that ApoB appeared from fraction 8 onwards, corresponding to low density lipoprotein particles. ApoA1^+^ particles appeared from fractions 9 onwards, contrasting with our own data, in which they were identified from fraction 6 onwards using either UF preconcentration step. Inherent differences in the content and viscosity of blood plasma and cell culture medium, as well as acknowledged variations in the abundance of lipoproteins would likely account for the observed inconsistencies in ApoA1^+^ fractions between our own study and that of Warnier et al. However, this finding highlights the need for EV isolation methods to be optimised depending on the starting material.

When applying SEC for the isolation of EVs, it is often necessary to combine it with a sample pre‐concentration step. This becomes essential when applying SEC for the isolation of EV from larger sample volumes or if attempting to scale up EV therapeutics. Sample concentration can be simply achieved using a UF column (Benedikter et al., 2017; Guerreiro et al., 2018; Mol et al., 2017; Nordin et al., 2015; Patras et al., 2021; Vergauwen et al., 2017). UF has also been applied independently of SEC for EV isolation (Cappione et al., 2014; Kornilov et al., 2018; Parimon et al., 2018). However, this will inevitably reduce the specificity of the technique and the purity of the EV preparation. The efficiency and specificity of UF is likely to be dependent on the composition of the UF membrane (e.g., cellulose, cellulose triacetate [CTA], polyethersulfone [PES] or modified nylon) and the MWCO applied (Vergauwen et al., 2017). To the best of our knowledge, no study has determined the effects of UF column pre‐concentration on EV recovery from SM models when combined with SEC. In the present study, we identified that different UF materials had an impact on EV recovery, as shown by variations in tetraspanin profiles and sample purity ratios (Figures 2 and 3). Samples obtained after Amicon pre‐concentration were enriched in CD9, Alix and CD81, while TSG101 was more prominent in Vivaspin 2–10 preparations (Figure 3d,e). This is a highly relevant finding, since it suggests that the choice of UF column can have a considerable impact on the composition of the resulting EV preparation, thereby impacting the outcomes of EV biomarker studies. Notably, SEC‐only controls were absent from our experimental workflow, due to the fact that samples generated using SEC resulted in a dilute preparation that required concentration to permit further analysis. As such, we cannot comment on the utility of SEC for EV isolation from our SM model independently of UF. While any adhesion of EVs to the UF membrane could be mitigated through the application of detergents (K. Lee et al., 2003), their effects on EV permeability and bioactivity would need to be further evaluated as they have previously been shown to have differential effects on EV subpopulations (Osteikoetxea et al., 2015). In addition to membrane properties, UF devices typically have MWCOs ranging from 3kDA to 100 kDa, depending on the commercial supplier and device selected. Previous studies have typically utilised columns with a 10 kDa MWCO in combination with SEC, demonstrating enrichment in EV‐like particles in cell culture medium, blood plasma and urine (Benedikter et al., 2017; Vergauwen et al., 2017). A study by Vergauwen et al. concluded that regenerated cellulose Amicon filters with 10 kDa MWCO recovered significantly more EVs, with less than 40% recovery reported for PES Vivaspin 10 kDa and other membrane types (CTA and modified nylon) (Vergauwen et al., 2017). However, in this study it was not possible to differentiate the effects of filter membrane from those of the MWCO, and no validation of defined EV markers was presented. A study directly comparing UF MWCO on EV recovery reported that a 100 kDa pore size was more effective at recovering EVs than 10 kDa cut‐off (Guerreiro et al., 2018). However, it should be noted that EV characterisation was minimal and based only on size (60–140 nm) and the expression of the CD9, which cannot be used as a specific marker for EVs and has been shown to be differentially expressed depending on EV source (Kowal et al., 2016). To the best of our knowledge, just one other paper has applied a UF pre‐concentration from C2C12 myoblast cells for SM‐EV isolation (Coenen‐Stass et al., 2019). In this instance, Coenen‐Stass et al. applied PES membranes (Vivaspin filters) with a MWCO of 10 kDa, observing SM‐EV recovery and TSG101 enrichment. However, fraction selection was based non‐specifically only on total protein presence (as measured by absorbance), where no clear separation between the EV and non‐EV components could be observed. Our research looked specifically at the effects of UF pre‐concentration on the recovery of SM‐EVs, comparing 100 kDa regenerated cellulose (Amicon) and PES (Vivaspin) filter membranes. We applied 100 kDa columns in the present study to eliminate major proteins present in the FBS and cell culture media (largest common protein identified in FBS being complement C3 at 187,135 Da) while retaining the EV fraction (Pisani et al., 2017). This results in further purification of the EV fraction and ensures our protocol is amenable to therapeutic EV production as well as diagnostic applications. It also makes the protocol applicable for the purification of EVs in more complex biofluids such as blood where proteins such as albumin are highly abundant. In conclusion, this portion of our data emphasises the need for the validation of EV isolation protocols that apply UF devices to ensure the translatability and reproducibility of findings when applied to the increasing number of EV studies in SM.

Finally, outcomes from the present study have validated a protocol (Amicon, fractions 1–5) for the isolation of SM‐EVs with increased purity and the absence of lipoprotein contaminants (ApoA1^+^ and ApoB^+^ positive particles). Size distributions of particles recovered using both UF filters aligned with those previously documented for small EVs isolated by SEC (50‐300 nm) (Coenen‐Stass et al., 2019; Estrada et al., 2020; Kalra et al., 2013; Sidhom et al., 2020) and other cell types, likely representing a mixture of small EVs comprising of exosomes and microvesicles (Askeland et al., 2020; Johnsen et al., 2019; Kornilov et al., 2018; Kratzer et al., 2016). The reduction of lipoproteins observed through the selection of Amicon fraction window 1–5 (Figures 4 and 5) did not appear to compromise the presence of EV material. Although the total number of particles was reduced (from 8.42 × 10^8^ to 4.85 × 10^8^ particles/mL, *p* < 0.05), sample purity, as indicated by the PTP ratio, increased (from 129 to 140, *p* > 0.05) and lipoprotein contamination was practically eliminated (1.4%, Figures 2 and 5). Although we acknowledge total lipoprotein elimination is challenging regardless of the method applied, this UF + SEC protocol reduced their presence to the minimum. A previous study has described how combining fewer fractions could increase overall sample purity, at the expense of EV yield (Ter‐Ovanesyan et al., 2021). Although, sample purity in this example was based on the presence of total free protein and no specific assessment of lipoproteins or other contaminants was included. Importantly, no obvious variations in the presence of tetraspanin positive EVs were encountered by reducing the fraction window to eliminate lipoprotein content, with changes in tetraspanin profiles resulting only from the type of UF column used (Figures 3, 4 and Supplementary Figure S2). Of note, variations can also be observed in the profiles of tetraspanins depending on the sensitivity and specificity of the method of analysis used, with flow cytometry‐based analysis only able to quantify externally expressed epitopes of transmembranal tetraspanin proteins. Lastly, in Figure 4 we provide a general overview of our study outcomes, with protocols for enhancing the purity (red stream) and total recovery (blue stream) of EV preparations depending on the objectives of a given research question. We also propose that alternative assessments of EV purity are considered in addition to simple protein and particle measurements, with calculations of tetraspanin enrichment and lipoprotein inclusion providing a more informative indication of sample purity until additional SM‐EV specific markers can be identified.

## 5 CONCLUSIONS

**FIGURE 6:**
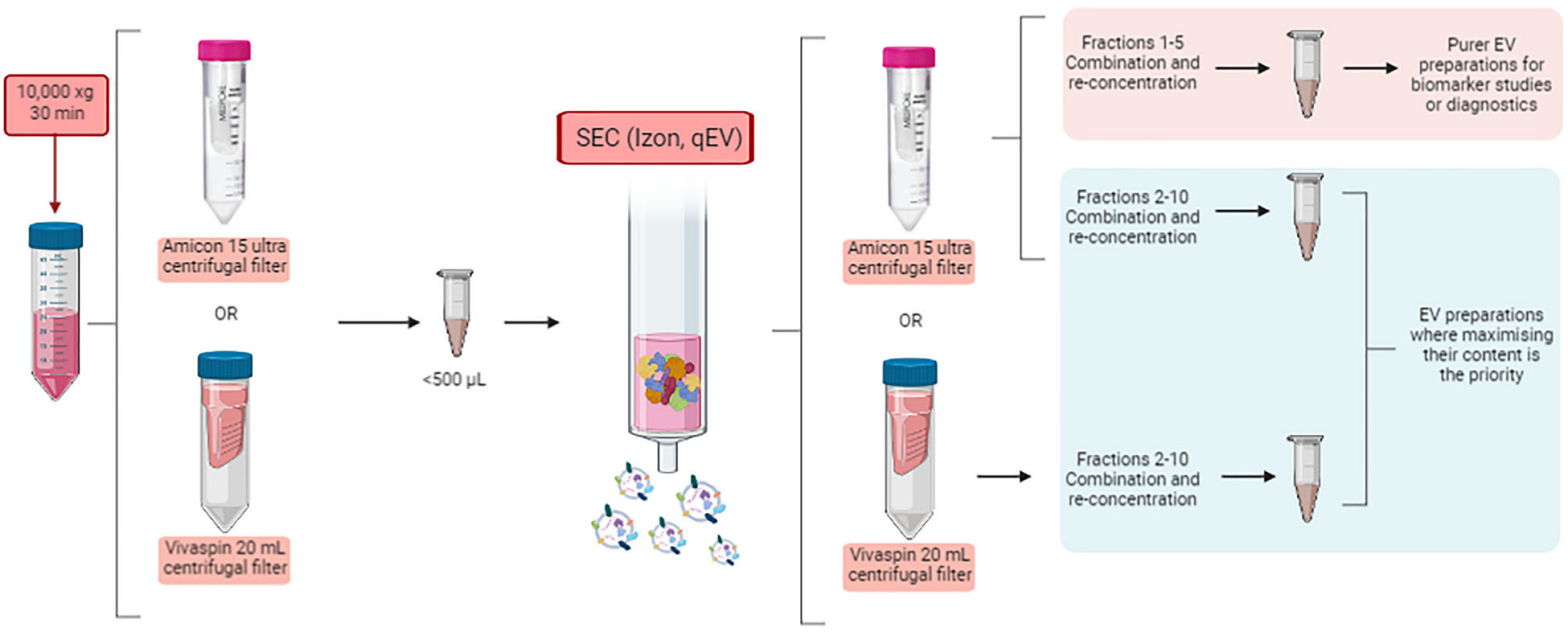
Workflow summary of the isolation of myoblast derived EV‐like particles when combining UF and SEC. Both isolation methods have their own merits, but their application needs to be differentiated. Amicon pre‐concentration and combination of fractions 1–5 could be more applicable to studies where lipoprotein contamination could be an issue, such as biomarker studies or diagnosis. However, both filtration protocols and combination of fractions 2–10 could be more suitable to maximise the EVs concentration on the preparation when working with samples where purity is not the main aim. (Created using Biorender (https://biorender.com/).

This study examined the application of two SEC + UF protocols for the recovery of EV preparations from an SM model. We identified crossover between EVs and lipoprotein components in standard SEC preparations. Reducing the fraction window to eliminate lipoproteins was shown to have no significant effect on EV recovery, as evaluated by the presence of common tetraspanin markers. However, when combining SEC with UF, the choice of UF column had a considerable impact on EV recovery, with differential marker profiles reported between Amicon and Vivaspin columns with identical MWCOs. As such, we emphasise the choice of UF column can have a significant impact on EV profile that could impact downstream applications. Outcomes from this study provide ability to clearly separate EVs from lipoproteins that could compromise downstream profiling and lead to invalid biofunctional conclusions. Our methodology has the potential to be translated into models of increasing complexity, to test and eliminate the presence of lipoproteins that could impact downstream biomarker and therapeutic studies. Lastly, there is the opportunity to apply these more stringent isolation criteria to validate the presence of circulating SM‐EV markers such as SGCA and luminal miRNA markers (including miR‐1, miR‐133a, miR‐133b, and miR‐206) that have been implicated as important regulators of tissue regeneration (Mytidou et al., 2021) (Figure 6).

## AUTHOR CONTRIBUTIONS

Experimental work, data analysis and manuscript preparation were performed by María Fernández‐Rhodes. Bahman Adlou collaborated in the data analysis. Soraya Williams collaborated in experimental work. Aveen R. Jalal was implicated in manuscript editing. **Owen G. Davies** was responsible for study conception, data analysis and manuscript editing. Mark P. Lewis contributed to manuscript editing. A first draft of the manuscript was written by María Fernández‐Rhodes and all authors commented on previous versions of the manuscript. All authors read and approved the final manuscript. **María Fernández‐Rhodes**: Conceptualization; Data curation; Formal analysis; Investigation; Methodology; Validation; Writing‐original draft; Writing – review & editing. **Bahman Adlou**: Data curation. Soraya Williams: Methodology; Validation. **Rebecca Lees**: Investigation; Resources. **Ben Peacock**: Investigation; Resources. **Dimitri Aubert**: Resources. **Aveen R. Jalal**: Writing – review & editing. **Mark P. Lewis**: Conceptualization; Funding acquisition; Project administration; Supervision; Writing – review & editing. **Owen G. Davies**: Conceptualization; Data curation; Formal analysis; Funding acquisition; Investigation; Methodology; Project administration; Resources; Supervision; Validation; Writing – original draft; Writing review & editing.

## CONFLICT OF INTERESTS

The author(s) declared no potential conflicts of interest with respect to the research, authorship, and/or publication of this article.

## CONSENT FOR PUBLICATION

Not applicable.

## Supporting information

Supplementary Table 1: Western Blot antibody guide for targeted proteins.
Supplementary Figure 1: Supplementary analysis on the individual 30 fractions after SEC isolation. (A) Grouped dot plot of 45 particle concentrations per fraction (15 x 3 repeats) between Vivaspin and Amicon.
Supplementary Figure 2: ExoELISA analysis.
Supplementary Figure 3: TEM images of SM‐EV collected after different UF+SEC protocols.

## Data Availability

The datasets generated during and/or analysed during the current study are available from the corresponding author on reasonable request.
