# Supplementary material for: Defining the influence of size‐exclusion chromatography fraction window and ultrafiltration column choice on extracellular vesicle recovery in a skeletal muscle model
Source: J Extracell Biol. 2023 Apr 21;2(4):e85. doi: 10.1002/jex2.85 (PMC11080914; doi:10.1002/jex2.85)
Supplement: Supplementary file 1 — Supplementary Table 1: Western Blot antibody guide for targeted proteins. Supplementary Figure 1: Supplementary analysis on the individual 30 fractions after SEC isolation. (A) Grouped dot plot of 45 particle concentrations per fraction (15 x 3 repeats) between Vivaspin and Amicon. Supplementary Figure 2: ExoELISA analysis. Supplementary Figure 3: TEM images of SM‐EV collected after different UF+SEC protocols. [file JEX2-2-e85-s001.docx]

# Defining the influence of size-exclusion chromatography fraction window and ultrafiltration column choice on extracellular vesicle recovery in a skeletal muscle model

María Fernández-Rhodes^1^, Bahman Adlou^1^, Soraya Williams^1^, Rebecca Lees^2^, Ben Peacock^2^, Dimitri Aubert^2^, Aveen R. Jalal^1^, Mark P. Lewis^1^, Owen G. Davies^1^

^1^School of Sports, Health and Exercise Sciences, Loughborough University, Loughborough, Leicestershire, UK

^2^NanoFCM Co., Ltd, Nottingham, Nottinghamshire, UK

Corresponding author: o.g.davies@lboro.ac.uk

# **SUPPLEMENTARY FIGURES**

ExoELISA ULTRA CD63 AND CD81

CD63 and CD81 levels were quantified using ExoELISA-ULTRA kits (EXEL-ULTRA-CD63-1/ EXEL-ULTRA-CD81-1, System Bioscience, UK). Equal volumes of EV preparations were immobilised onto the wells of a 96-microtitre plate and the assay carried out according to the manufacturer’s instructions.

**Supplementary Table 1: Western Blot antibody guide for targeted proteins.** We included information about dilutions, source, supplier and codes and secondary antibody dilutions used in the results represented in this report.

| Primary Antibody | Source | Dilution | Supplier | Secondary antibody | Dilution |
| --- | --- | --- | --- | --- | --- |
| Anti-Alix | Rabbit | 1:1000 | Santa Cruz (sc-53540) | Anti-Rabbit | 1:3000 |
| Anti-Annexin A2 | Rabbit | 1:2000 | Abcam (ab41803) | Anti-Rabbit | 1:3000 |
| Anti- TSG101 | Rabbit | 1:1000 | Abcam (ab30871) | Anti-Rabbit | 1:3000 |
| Anti-CD9 | Rabbit | 1:1000 | Abcam (ab92726) | Anti-Rabbit | 1:2000 |
| Anti-CD63 | Rabbit | 1:1000 | Abcam (ab216130) | Anti-Rabbit | 1:3000 |
| Anti-Calnexin | Mouse | 1:1000 | Abcam (ab22595) | Anti-Mouse | 1:3000 |
| Anti-ApoA1 | Rabbit | 1:1000 | Abcam  (ab20453) | Anti-Rabbit | 1:3000 |
| Anti-ApoB | Rabbit | 1:1000 | Novus Biological (NB200-527) | Anti-Rabbit | 1:3000 |


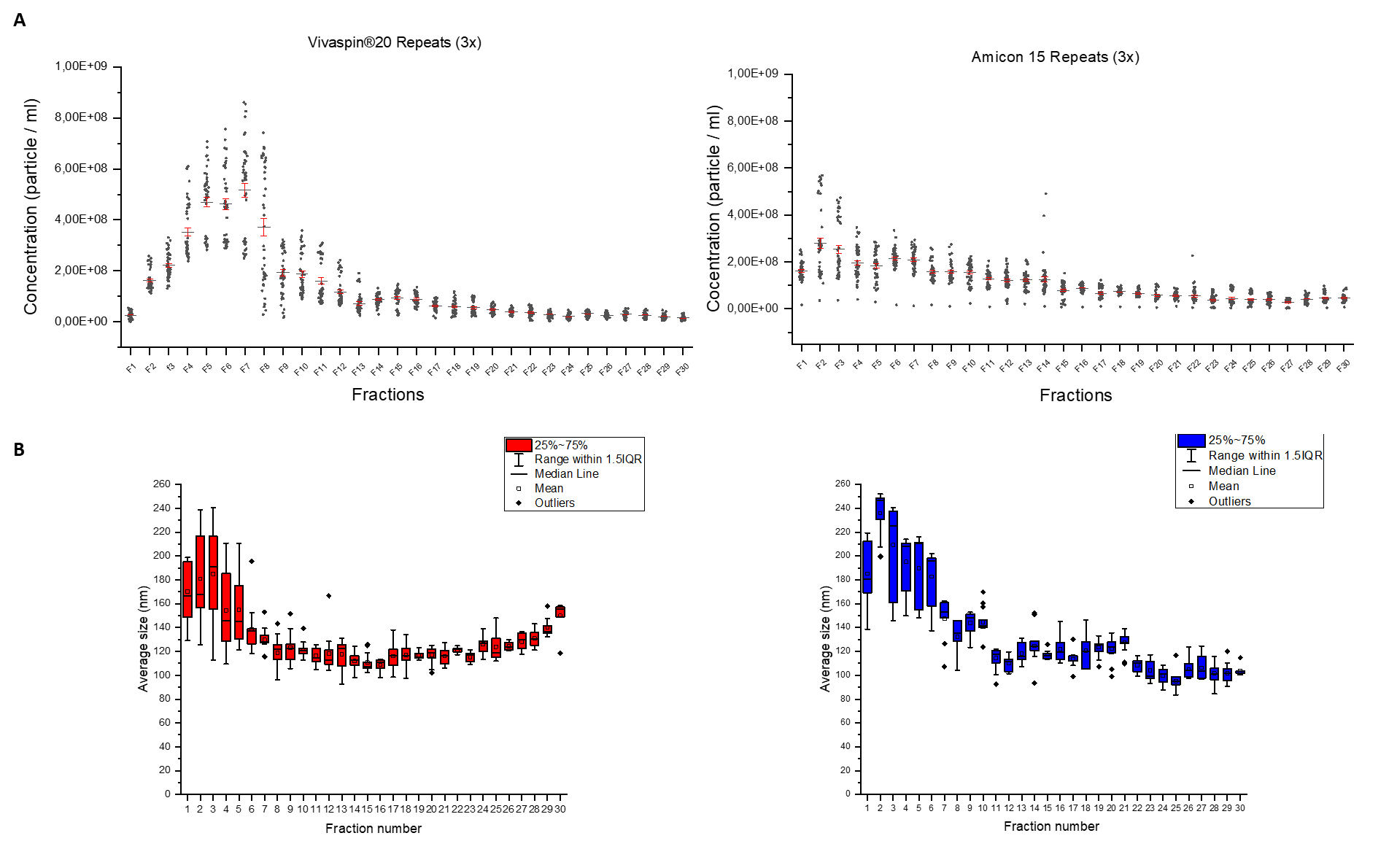


**Supplementary Figure 1: Supplementary analysis on the individual 30 fractions after SEC isolation. (A) Grouped dot plot of 45 particle concentrations per fraction (15 x 3 repeats) between Vivaspin and Amicon.**  Vivaspin fractions =<14: 2.43E+08 ± 6.35E+07 and fractions >=15: 4.27E+07 ± 8.08E+06. Amicon fractions =<14: 1.76E+08 ± 3.45E+07 and fractions >=15: 5.39E+07 ± 6.40E+06. **(B) Target fractions size distributions for each filter.** Amicon (red) and Vivaspin (blue) Amicon average size distribution: 158.15 ± 19.33 nm vs. Vivaspin’s 163.41 ± 30.12 nm.


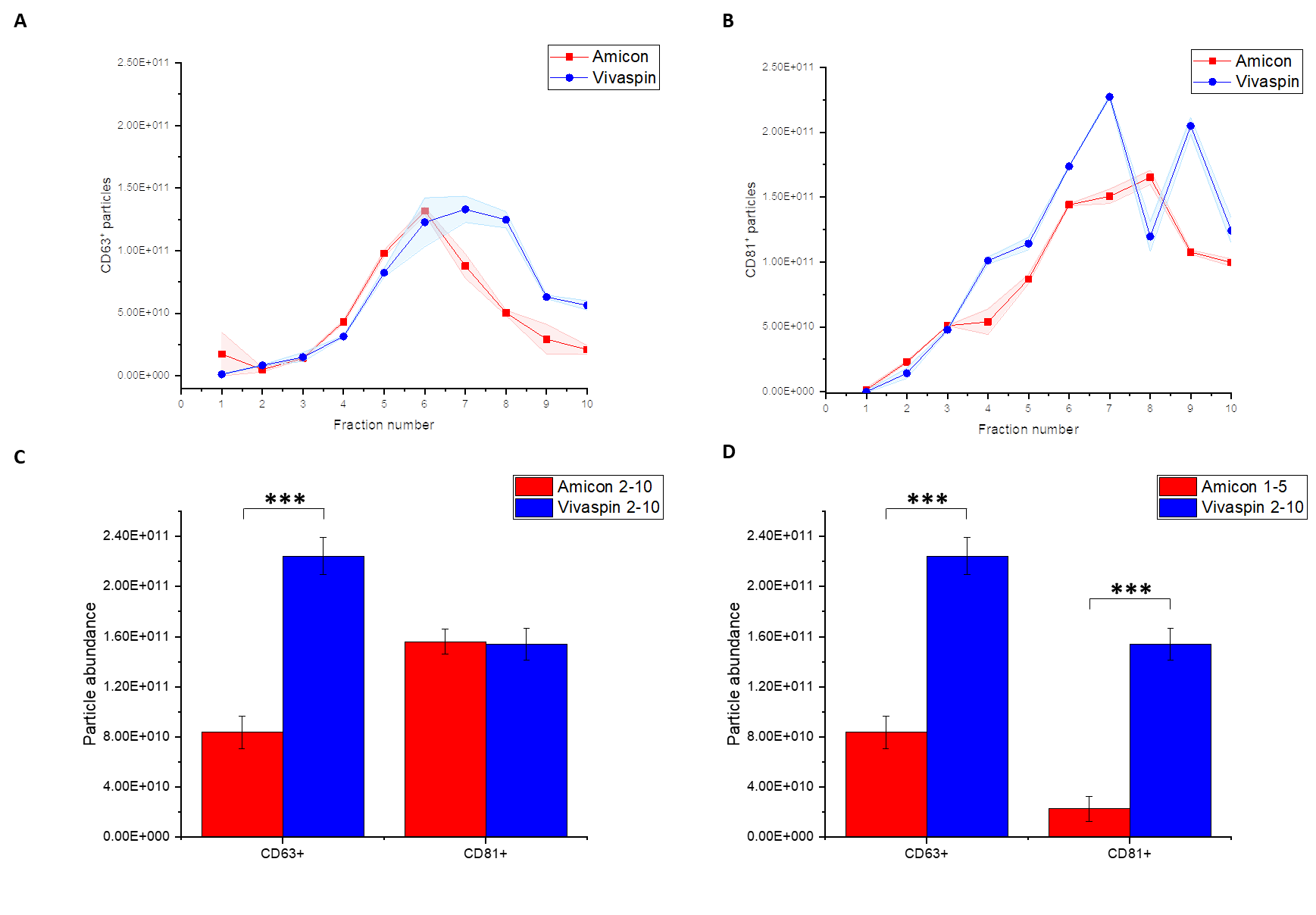


**Supplementary Figure 2:** **ExoELISA analysis.** (A) Presence of CD63^+^ particles in Amicon (red) and Vivaspin (blue) over fractions 1 to 10 (B) Presence of CD81^+^ particles in Amicon (red) and Vivaspin (blue) over fractions 1 to 10 (C) CD63^+^ and CD81^+^ particle abundance in final preparations after Amicon or Vivaspin pre-concentration, combining fractions 2 to 10 (D) Presence of CD81^+^ particles in Amicon (red) and Vivaspin (blue) over fractions 1 to 10 (C) CD63^+^ and CD81^+^ particle abundance in final preparations after combining fractions 1 to 5 after Amicon pre-concentration and fractions 2 to 10 after Vivaspin pre-concentration ( ***;p<0.001).


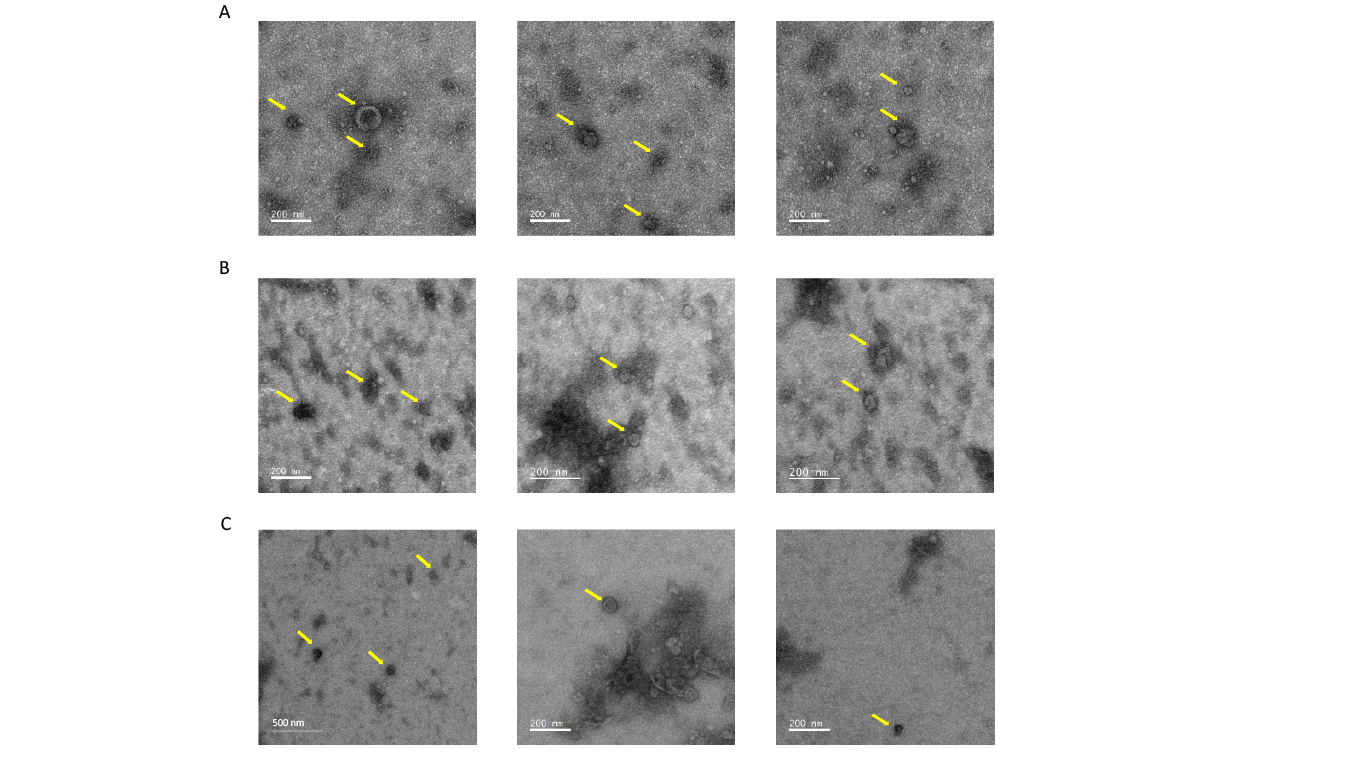


**Supplementary Figure 3: TEM images of SM-EV collected after different UF+SEC protocols.** (A) Images panel for samples obtained after Amicon UF formed by fractions 2-10 (B) Images panel for samples obtained after Vivaspin UF formed by fractions 2-10 (C) Images panel for samples obtained after Amicon UF formed by fractions 1-5.
